# Illinois Charitable Eye and Ear Infirmary

**Published:** 1879-02

**Authors:** F. C. Schaefer

**Affiliations:** Assistant Aural Surgeon


### Clinical Reports.

### Article VI.

Illinois Charitable Eye and Ear Infirmary. Cases presented at Dr. Jones' clinic, and reported by F. C. Schaefer, M. D., Assistant Aural Surgeon.

*Eczema of Left Auricle and Auditory Canal.*

This child, Mary W., aged 6 years, has an eruption located chiefly in the meatus externus of the left ear, which is exceedingly painful to the touch. It presents the appearance of a crusted skin weeping a yellowish fluid, and gives rise to more or less pruritus. The eruption has probably existed several months ; no accurate history can be obtained regarding it. This disease is known as eczema madidans, here in the sub-acute stage.

Eczema appears in the acute, sub-acute and chronic forms. The acute is the most common, and occurs usually in poorly fed female children, and in those who have inherited a strumous diathesis. It is sometimes brought on in children by uncleanliness or by their picking the ears. Occasionally the disease is met with in the adult, resulting from the application of irritating substances to the part. This pathological condition may be idiopathic, or may occur in the auricle by reason of its contiguity to the disease in adjacent parts. In this case we find it invading the auditory canal, encroaching slightly upon the auricle.

Eczematous eruptions, as you are aware, are, by some, classed among vesicular skin diseases. They usually appear first in the form of vesicles, but in many instances, especially about the ears, the vesicles coalesce at their circumference ; their liquid contents, at first clear, become semi-purulent, and exuding from the vesicles dry into a scab.

The treatment varies with the stage of the affection. In the acute form sedatives to the part are indicated. Burnett and Roosa use for local application :

| | |
|---|---|
| Flor. zinci | 8 |
| Alum pulv | 30 |
| Amyli pulv | 30 |

The powder is to be dusted thoroughly over the diseased part.

Should there be a burning sensation present, with tendency to inflammation, astringent lotions may be used, the eruption to be disturbed as little as is consistent with effectual treatment. The air to be excluded as much as possible.

The sub-acute stage calls for about the same treatment, with the addition of moderate stimulation with benzoated oxide of zinc ointment.

In the chronic form, emollients are useful to soften the scales, and more stimulating applications are recommended, as the citrin with zinc ointments, and such others as may suggest themselves to your minds. Should the patient present a cachectic physiognomy, constitutional treatment will aid in the result. Cod liver oil and various ferruginous preparations are very serviceable in some cases, and Fowler's solution in others. Cleanliness always plays an important factor. You will however be governed in your therapeutic measures by the individual case.

*Probable Syphilitic Gumma in the External Auditory Canal.*

Miss S., aged 24. The external auditory meatus presents a conical tumor about 3 Mm. in height and 2½ Mm. wide at its base, on the posterior wall of inner third of the canal. The tumor has been present a number of months. Upon first examination several weeks ago, it seemed like an exostosis, but during the last few days it has become considerably inflamed and softened, and is now quite tender. The patient is inclined to resist the touch of a probe. You also notice in front of the tragus an indurated excrescence which seems to be cartilaginous ; this appeared more recently and has developed rapidly. Exostoses are usually not painful, and therefore this tumor may be regarded as doubtful and somewhat suspicious. The patient gives no specific history, yet the growths are indicative of syphilis, doubtless inherited, and it will be well to place her upon tonic and alterative treatment, with the expectation of causing the tumors to disappear.

Iodide of potassium and tinct. cinchona were prescribed.

*Impacted Cerumen and Polypus in the External Meatus of one Ear, with Inflammation of both Tympana.*

Attention was called to Miss M., aged 20. The patient said her hearing had been defective for several years. Has more or less tinnitus aurium now. States that the right ear only is affected. Examination with the otoscope reveals a milky, turbid appearance of the membrana tympani, indicative of a thickening of the part due to an inflammatory process. The triangular light spot present in the healthy membrane is here absent, because the thickening has changed its angle with the external auditory canal, and its turbidity has diminished its reflecting power.

The patient has a peculiar intonation of the voice in talking; she speaks in a high key. This condition of the voice would of itself indicate to an otologist that there is a defect in the auditory apparatus, which has lasted a long time. This manner of talking suggests to us that the other ear is also involved. We shall see. When a watch is placed a few centimeters from the ear she can not hear it. If you now look through the otoscope, you will see a dark, dry substance, with spots like flakes of bran — dry, epithelial scales scattered over its surface. This mass is impacted cerumen and desquamated skin; (the impacted mass is removed). You see, gentlemen, we have here a hard substance the size of a bean; so hard that the instrument elicits a grating sound as we rub it. The usual procedure for removing these accumulations is to syringe the ear with alkaline water. By repeated syringings the mass is softened and finally washed out, but as we wished to examine this case further to-day, a quicker method was resorted to, which, as you saw, consisted in lubricating the meatus with vaseline and seizing the cerumen with the angular forceps, extracting it by a gentle, vibratory motion. (Introduces otoscope.) We now detect upon the posterior wall of the auditory canal, not far from the membrana tympani, a red projection, presenting a raspberry-like appearance, forming a half sphere, having no pedicle; this is a polypus, a result of suppurative inflammation of the tympanum. I shall now proceed to remove it with these forceps. There, the greater part has been extracted. What remains forms an irregular surface. To remove this, chlor-acetic acid will be applied. The patient winces under the treatment. The application of caustics is of course painful. Chlor-acetic acid is used because it can be better controlled than nitric acid; it does not manifest the same disposition to spread, and its caustic property is destroyed by diluting it with water, thus stopping its action. A view of the membrana tympani can now be obtained, and reveals the fact that it is even more opaque than the other, presenting a slight perforation near its base.

This case is instructive in that it should teach you the necessity of following up the objective as well as the subjective symptoms, and shows that although there may be present in the meatus a hard, dry accumulation, a granulating surface beneath it, may be found after removing what has practically become a foreign substance.

*Mastoid Periostitis, Suppuration.—Eczema.*

This little girl, less than two years of age, was brought here two days ago suffering with periostitis extending from the mastoid process upwards and forwards until the swelling has reached the superior border of the external auditory meatus. As the inflammation was of a suppurative character, poulticing was advised, and to-day we detect fluctuation. It will be necessary to cut deep, and to make a free opening. Observe how the introduction of the lancet to the bone above the auricle is followed by the escape of pus. There is evidently a sinus extending in the direction of the mastoid process. The poultices should be applied several days longer. You see the strumous diathesis in this child. Such children are subject to enlargements of the glands and periosteal inflammations. Frequently eczematous eruptions are found upon the skin, as here manifested, over the opposite ear.

*Chronic Non-Suppurative Inflammation of Both Tympana, with Calcareous Deposit in the Membrana Tympani.*

Mr. B., æt 57, entered the infirmary May 11, 1878; hearing had been more or less defective for three years, supervening upon catarrhal inflammation of the pharynx; he had tinnitus aurium in both ears. At the present time there is no tinnitus, and his hearing is almost perfect. The professor introducing the otoscope invited the gentlemen present to look at the membrana tympani, saying the membrane presents almost a normal appearance with reference to its transparency. The triangular light spot is distinctly visible in the lower anterior quadrant, indicating that the membrane is but slightly thickened by the product of inflammation.

Changing the position of the otoscope you now see a peculiarly opaque, white spot, near the upper border of the drumhead, somewhat papular in appearance; this is a calcareous deposit, a not infrequent accompaniment of old age. It does not necessarily compromise the function of hearing. It is desirable that every member of the class should see the membrane in this case, in order that he may recognize the difference between healthy and diseased membranes of the drum as they come under notice, for it is essential to know, first—*what to look for*, and second—to *interpret what is seen.*

The triangular light spot is the first landmark, so to speak, that is looked for in examinations of this part. This triangle, or pyramid of light, in the lower anterior quadrant of the membrana tympani, is always present when the membrane is in a healthy state; so you see it is very important that you should recognize it when present, and the condition of the parts in its absence. The light spot is produced by a combination of three causes:

*First.* The lustrous epithelium of the healthy membrane, making it a good reflector.

*Second.* The peculiar angle formed by the junction of the membrane with the external auditory canal, and

*Third.* The funnel-shape of the membrana tympani.

Any deviation from the natural brilliancy of the membrane indicates a departure from the healthy condition.

